# The challenges of theory-software translation

**DOI:** 10.12688/f1000research.25561.1

**Published:** 2020-10-02

**Authors:** Caroline Jay, Robert Haines, Daniel S. Katz, Jeffrey C. Carver, Sandra Gesing, Steven R. Brandt, James Howison, Anshu Dubey, James C. Phillips, Hui Wan, Matthew J. Turk

**Affiliations:** 1 University of Manchester, Manchester, UK; 2 University of Illinois at Urbana-Champaign, Urbana, USA; 3 University of Alabama, Tuscaloosa, USA; 4 University of Notre Dame, Notre Dame, USA; 5 Louisiana State University, Louisiana, USA; 6 University of Texas at Austin, Austin, USA; 7 Argonne National Laboratory, Argonne, USA; 8 Pacific Northwest National Laboratory, Richland, USA

**Keywords:** scientific software, research software engineering, research software, scientific computing, high performance computing, scientific software development

## Abstract

**Background**: Software is now ubiquitous within research. In addition to the general challenges common to all software development projects, research software must also represent, manipulate, and provide data for complex theoretical constructs. Ensuring this process of theory-software translation is robust is essential to maintaining the integrity of the science resulting from it, and yet there has been little formal recognition or exploration of the challenges associated with it.

**Methods**: We thematically analyse the outputs of the discussion sessions at the Theory-Software Translation Workshop 2019, where academic researchers and research software engineers from a variety of domains, and with particular expertise in high performance computing, explored the process of translating between scientific theory and software.

**Results**: We identify a wide range of challenges to implementing scientific theory in research software and using the resulting data and models for the advancement of knowledge. We categorise these within the emergent themes of design, infrastructure, and culture, and map them to associated research questions.

**Conclusions**: Systematically investigating how software is constructed and its outputs used within science has the potential to improve the robustness of research software and accelerate progress in its development. We propose that this issue be examined within a new research area of theory-software translation, which would aim to significantly advance both knowledge and scientific practice.

## Introduction

Software has transformed scientific practice, creating new forms of analysis and representation, and enabling research or thinking that was not previously possible. The growing use of computation has also added significantly to the complexity of conducting research. 

The process of representing, precisely, a scientific entity, method, or system in software is extremely challenging. Having sufficient accuracy is paramount: the more the implementation deviates from the concept it is intended to represent, the lower the value of the resulting knowledge. Verification and validation—commonly referred to as V&V—have the potential to give a level of confidence that the software is both a correct representation of the theory and free of defects ^1^. Verifying the accuracy of a scientific artefact is difficult, however, as there may not be an oracle against which to test it. The artefact may also pass a set of tests, yet still contain errors that have been introduced, unnoticed, during the engineering process. Validation of the artefact—being sure it is a true representation of the theory—is even more challenging. 

The fact that we are able to design and build computational systems does not mean we fully understand them or what they do, or that they do exactly what we want them to do; the proliferation of defects found during the lifetime of any software system illustrates how difficult it is to accurately predict how software will behave at runtime. Huge progress towards system reliability has been made through formal approaches to software verification, and comprehensive tooling exists to assist with many aspects of programming, from detecting code smells (e.g., duplicated code, highly coupled entities, or high cyclomatic complexity), to monitoring test coverage. In spite of this, defects remain a significant problem. 

In addition to addressing the general difficulties common to all software development projects, research software must represent, manipulate, and provide data for complex theoretical constructs. Such a construct may take many forms: an equation, a heuristic, a method, a model; here we encapsulate all of these, and others, in the term *theory*. In the process of mapping a theory to a programmatic or software-based implementation, defects may occur at a number of points:

- The *science* is wrong: The theory itself may contain defects, which are discovered through the process of trying to represent it computationally.
- The *software* is wrong: The way in which the code is written may contain defects—although it is possible to translate from theory to implementation, the chosen form is not appropriate.
- The *translation process* incurs loss or ambiguity: whilst it may be straightforward to represent a theory verbally or mathematically, it may be difficult to represent it computationally— “Mathematics provides a framework for dealing precisely with ‘what is.’ Computation provides a framework for dealing precisely with ‘how to”’ ^2^.

All of these situations, and the last in particular, interact to make the process of conducting computational research complicated and defect-prone, resulting in a human-machine translation gap.

Within science, any form of defect or unreliability is highly problematic: if the software does not behave as desired or anticipated, the results may not stand. At present, we lack any formal means of explaining how and why an implementation differs from a concept in unanticipated ways. Whilst a mismatch may be due to obvious limitations of the representation (e.g., ﬂoating point rounding errors), the way in which an implementation is constructed can also result in inaccuracies that were not apparent at the time they were created. Understanding these issues, via empirical research, is vital to ensuring the accuracy and validity of research software.

Compounding the difficulties of formally translating between theory and software are the many cultural and organisational factors that add further challenges to the process of building research software and using its results to advance science. These include the bespoke and highly dynamic nature of research software, the funding model, the academic hierarchy and career structure (in particular the difference in status accorded to domain and software specialists), the difficulties communicating in large scientific teams, and the pressures exerted by the current publication model ^3^.

In this paper, we provide evidence to motivate the systematic investigation of the Theory-Software Translation process. We achieve this through analysis of the discussion sessions that occurred at the Theory-Software Translation Workshop held in New Orleans ^4^ in February 2019, which explored in depth the process of both instantiating theory in software—for example, implementing a mathematical model in code as part of a simulation—and using the outputs of software—such as the behavior of a simulation—to advance knowledge.

In the Methods section, we describe the workshop format and goals, its participants, and the process used to collect and systematically analyse data from the discussion sessions. In the Results section, we present the themes that emerged from the analysis, and map these to potential research questions. In the Discussion section, we compare our findings to those of other work in this area, in particular the earlier Code/Theory workshop that took place in the UK ^3^. We conclude by summarising the case for theory-software translation research, and proposing future activities that will lead towards establishing it as a new and fruitful domain.

## Methods

The workshop report ^5^ contains a full description of the event, including the agenda, participant list, talk titles and supplementary materials. Below, we describe the key details relevant to the analysis reported here.

### Format

The workshop started with an introduction from the organisers, which was followed by talks from the participants on their background and interest in the topic. The main part of the workshop consisted of a series of breakouts. The first, where participants were pre-allocated to groups to ensure each group had people with a mix of backgrounds, focused on defining the overall challenges of theory-software translation. Following a feedback session, and noting the themes that were starting to emerge, the organisers divided the next set of breakouts into groups that considered *training and culture*, *software design*, *software stack and tools*, and *miscellaneous* (to catch any issues falling outside the first three). Participants self-selected to join one of these groups for one session, and then moved to a different group for the next session. In each case, participants were asked to discuss the topic, list challenges, identify current successes, and indicate how we could make progress. A final plenary session considered the prospects for Theory-Software Translation as a research area, and considered next steps.

### Participants

Workshop participants comprised 20 experts in the field of High Performance Computing and researchers interested in the process of research software engineering. Nine participants were employed at the time at US national laboratories, and 11 at US or UK universities. Three participants were academics whose main focus was studying the process of research software engineering. The rest were involved in research software engineering or the management thereof, with an interest in the idea of theory-software translation, and a desire to improve the process of research software engineering. A full list of participants and their talk materials can be found in the workshop report and on the website ^4, 5^.

### Analysis

During the breakout sessions, groups kept a record of their conversation, transcribing as much of the discussion as possible, and then summarising key points at the top of the document. There were three breakout sessions, each with four groups, resulting in 12 discussion documents. The first breakout session was split into two parts: in the first part, people wrote notes; in the second, the topic document was given to a different group who added to and commented (using the ‘add comment’ functionality) on the contents. Because common topics arose across groups and sessions, the breakout notes were analysed as a single corpus. Two of the authors (CJ and RH) performed a thematic analysis, with CJ coding the full set of discussion notes and generating initial themes, RH reviewing these and cross-checking with the discussion notes, and both iteratively refining the final set. Formal analysis software was not used for this process; instead the text was collated into a single document, and then parts were grouped together and labelled under subheadings, following familiarisation with the data.

Following this initial analysis, all workshop attendees were invited to review and comment on the results, and 13 subsequently endorsed the output as authors of the workshop report ^5^. None of the participants raised any issues with regard to the results of the analysis, or expressed a view that they were unrepresentative. The final stage of the analysis is presented in this paper, in which the authors (a self-selecting subset of the workshop attendees) have collectively further refined the sub-themes within this document via working on the manuscript draft together, providing more detail and adding examples.

### Consent

The discussion documents are collectively owned by the workshop participants. During the workshop, it was agreed verbally that these would be analysed and written up in a report, which all participants would be invited to author. This model of collective data ownership and knowledge production had previously been used successfully in the Code/Theory Workshop ^3^.

## Results

Three overarching themes emerged during the analysis, aligning to the challenges of *design*, *infrastructure*, and *culture*. We explain these below and describe the key areas of research identified within each, framed as open questions.

### Design

Participants considered *design* in terms of software design, research design, and the way in which the two interact. Topics covered included the extent to which it is possible to separate concerns, whether theory should be ‘readable’ from software, and potential techniques for evaluating and improving the design process.

**Can/should we separate concerns?** In an era of growing complexity in research models and questions, translating scientific theories to software in a reliable way is becoming increasingly difficult. One perspective on the process is that of moving from ‘science’ to ‘equations to be solved’ to ‘computational algorithms/numerical analysis’ to ‘computer science/software engineering’. (See Babuska and Oden ^1^ for a formal description of this process and these domains.) Each of these is a discipline in its own right, and each is complex. There was a view that it is not realistic for every scientist to understand all of these, and thus an informed ‘separation of concerns’ is crucial. Considering these parts of the process independently also allows each individual in a research team to focus on the aspect(s) for which they are most qualified. 

An alternative view was that concerns cannot always be separated within computational research, from both a theoretical and a practical perspective. At present, a paper and a code are separate things, but the boundaries are blurring. Jupyter notebooks are an example of documentation interspersed with executable code, but this approach is unlikely to be sufficient or scalable on its own. If the boundaries between publication and code increasingly overlap, then it becomes difficult to see where the theory ends and the software begins. Simply documenting the code by commenting it with the theory increases the maintenance cost of the code and risks the two becoming out of sync. Code marked up with the wrong theory is worse than useless, even dangerous, so it is important to be able to verify that the code and theory are consistent. Ince *et al.* ^6^ argue for the necessity of source code provision along with papers, citing research showing poor effectiveness of specifications in producing equivalency across implementations ^7^.

Another example of how boundaries are becoming blurred by the introduction of computational methods, is the fact that code and theory are increasingly developed alongside each other. Although it is natural to think (and is most often indeed the case) that one needs to formulate the equations and *then* apply computational algorithms to obtain the numerical solutions, the formulation of the equations can be affected by the choice of computational method. For example, the equations representing the physics behind a wave will be written for different quantities and hence take different forms, depending on whether a wave pattern is numerically described by a collection of discrete values sampled at selected locations, or the superposition of a number of Fourier modes.

**Should theory be readable from software?** There was considerable discussion about the extent to which it is possible to write research software in a way that maintains the essence and readability of the underlying theory. Software is highly complex, and can unintentionally obfuscate the theory it contains, particularly when it is optimized for high performance. Preserving a balance between readability (in terms of how easy it is to understand the code) and performance can be difficult. Optimizing code often makes it harder to understand, potentially obfuscating the theory that the software represents, and making it more difficult to reproduce, maintain and modify.

In an ideal project, mathematical concepts are contained in software components, offering reuse, support for testing, and a clear map to and from the underlying theory. Modular representation of theory is likely to be more readable and testable, but it would be interesting to investigate whether there are areas where this approach is not suitable.

A number of questions emerged from this discussion: Is there a particular design process that should be used for embedding theory within software such that it is readable? To what extent is it necessary for someone reading the software to understand the underpinning theory? When software is assembled from many components, each having their own theoretical foundations, what does this mean for conveying the overall theory underlying the whole? Is there value in an unoptimized, understandable version of a simulation serving as a reference implementation?

To facilitate theory-software translation in practical terms, domain norms and expertise may need to be taken into account. An example of this can be found in the US Department Of Energy’s effort to develop a new version of its Earth system model ^8^ for cutting-edge computational platforms. The final production code will be written in C **++** using Kokkos ^9^ for performance and portability. Because most climate scientists are trained in Fortran, a two-step approach is being used: the domain scientists develop their code in Fortran, then the computational scientists and software engineers take the Fortran code, translate it to C **++**, and then work on HPC performance. What are the benefits and trade-offs of introducing these further translation steps into the software development process?

**What are the effects of automation in programming?** In the future, code generators may offer a route to translating theory to software. This approach could do a better job of preserving information during implementation and lead to a higher order transformation, due to higher order input. It may allow for timely cross-code validation, where different theory comparisons are made, as it is less human-resource-intensive. This may also be a way to reduce human error (for example, one of the General Relativity solvers ^10^ in the relativistic astrophysics Einstein Toolkit ^11^ uses a Mathematica-based code generator called Kranc ^12^, as this work would otherwise be repetitive and error prone), although it should be noted that code generators, being software themselves, may also introduce defects. Recording the provenance of the code is important in understanding how theory is ultimately arrived at through software outputs. Does using a code generator obfuscate that provenance, or make it clearer?

**How can we evaluate the design process?** There are many ways of expressing theory in software. Gathering evidence for what works well would help to inform and refine the software design process. One approach to empirically examining software design is model inter-comparison, which is the process of comparing the results of different implementations of the same underlying theory, such as different climate models, and trying to understand the reasons for, and sources of, similarities and differences in model outputs. 

This is a technically challenging endeavour, and how to do it remains an open research question, but the results could provide an understanding of the efficiency and effectiveness of different implementations, and open up opportunities for code adaptation and reuse. Could we adapt existing codes to new paradigms? Domain specific languages (DSLs) are generally community specific at present. Could we make progress through merging or integrating them, at least where we can be reasonably certain that the models that they are representing are comparable? There is an explosion of tools and services across all domains. How can we tell if they are reliable? Would being able to compare them across domains help with the verification and validation of these tools?

**How can we better link domain science and computer science?** There appears to be a disconnect between computer science research and its deployment in scientific discovery; improving the linkage could lead to better science, and more efficient use of computing resources. There are many areas that require computer science research: new languages; more ﬂexible operators; code generation; code transformation; test generation. Theory-software translation research was recognised as having the potential to expose and contribute to these challenges.

### Infrastructure

Theory-software translation is not solely about mapping scientific constructs to algorithms, but rooted in and affected by a wider software and hardware infrastructure. This part of the discussion gave consideration to verification and validation, sustainability and portability, and how the uncertainty introduced by infrastructure might be recognised and measured.

**How should we verify results arrived at through computation?** There is currently no formally established, efficient means of verifying a software simulation, and as such this is an area that requires further attention. Where there is unexpected behavior in a simulation, both software and data provenance are crucial to knowing whether it is caused by a defect or highlights a discovery. Where there is a defect, how can we tell where it lies? Is it in the theory, or the mathematics, or the code? Knowledge is required, not just of the code and the theory, but of the full software stack, including the sequence of dependencies, and how the code is compiled or interpreted. 

The number of potential inputs to most codes is much larger than can be tested in its entirety. A further barrier to comprehensive test coverage is presented by the way in which some applications are configured—both at build-time and run-time. In large, ﬂexible codes features, methods and algorithms can be switched on or off, or swapped; how do we test all of these permutations and combinations of configurations to ensure that they do not interact with each other in unexpected ways? Is there a way we can express theory as a set of tests for code to pass, and ultimately automate test generation from theory specification?

**How should we address reproducibility and sustainability?** The importance of reproducibility within research is becoming increasingly recognised. The extent to which true reproducibility is possible in computational science is not clear, due to portability problems, continually changing technology and ‘software collapse’, where software stops working due to changes in underlying layers ^13^. Nevertheless, it was seen as important to strive to get as close as possible to this ideal, and also to work out practical ways of achieving something that approximates this. Having different teams trying to reproduce results, through multiple people running the same codes, could be useful in terms of verification and building knowledge. 

Whilst sustaining software for reproducibility is difficult and resource intensive, paradoxically, software almost always lives longer than planned, as (for example) adding features to a prototype is quicker and cheaper than engineering a new and robust code from scratch. What are the implications of this for theory-software translation? What are the effects on the software’s integrity, the way new theory must subsequently be implemented, and the results it produces? What are the issues caused by technical debt?

**What are the constraints posed by platforms and architectures?** Scientific software is generally going to be utilized on multiple generations of computational architectures, and the original developers of the software typically do not (and cannot) take this into account. Changing hardware impedes both portability and reproducibility. Build systems and supporting infrastructure also require maintenance, and any updates to these also have the potential to introduce defects. Where concepts or operations require workarounds to implement on current hardware—such as the representation of real numbers ^14^—the view was that we should ideally aim to change the hardware, rather than restrict the theory, while accepting that this is rarely possible.

We should also remain mindful that hardware, as well as software, can be an error source, as code that functions correctly on one platform may not on another, unbeknownst to the programmer.

**Measuring uncertainty in theory-software translation** Whilst theory is often exact, code has tolerances and approximations. Recognising this was seen as an important part of understanding and improving theory-software translation. One suggestion was to frame this issue in terms of implementation decisions introducing uncertainty. Rather than assuming, ‘this output is correct,’ would it be better to state, ‘there is x% chance some error has been introduced along the way, according to the architecture/code size etc., and therefore we should interpret the result accordingly?’ Could we develop diagnostics that verify the ‘health’ of the simulation, such that we could estimate the potential for defects caused by issues with code quality or age? There is also loss when moving between different stages of theory-software translation (theory, equations, algorithms, software). How can we measure this, and understand its effects?

### Culture

The environment in which theory-software translation takes place was recognised as a key inﬂuence on the process. Discussion relating to this topic covered collaboration, expectations, research environments and use of software engineering process.

**How can we foster a culture of collaboration?** Computational science, particularly that conducted in large projects, is necessarily interdisciplinary. The heavily domain-contextual specification of the problem and the deep technical knowledge required to implement solutions can lead to an initial communication barrier between domain scientists and computer/computational scientists. Embedding software engineers and applied mathematicians in research teams is a good way of facilitating communication, and there was discussion about what more could be done. One question was whether explicitly recognising the idea that software is a translation of theory might change the communication process. Could conversations across different roles be improved using this approach? The US Department of Energy’s Scientific Discovery Through Advanced Computing program ^15^ is an example of interdisciplinary efforts that directly engage computer scientists and applied mathematicians with the scientists of targeted application domains, with promising results. 

Implementing theory in code was viewed as different from implementing non-research software, especially where the requirements are concerned. A key issue was that it may not be possible to separate specification from design, a situation analogous to building an aircraft in ﬂight. Given this, there is a lack of clarity about the best way to approach requirements engineering within research projects. 

It was viewed as crucial to emphasise that software engineering is a core intellectual contribution to the research, not just a service. Close interaction between an application scientist and an applied mathematician can be helpful in designing the appropriate mathematical/numerical method. The discussion about the ‘separation of concerns’ within research software design extends to research software teams. Separating concerns too strictly may lead to different people concentrating on their own tasks, with their own goals and motivations, neglecting the overall picture. On the other hand, focusing on a particular aspect can provide better abstractions and more performant solutions. How do we balance these two pressures?

**What are the external expectations of the reliability of the software?** Validation, which was discussed extensively from a technical perspective, was also considered from an administrative/organisational perspective. Software may need to be considered as a scientific instrument that needs to be validated and/or calibrated. A current example of this is that in the UK, any software that collects patient symptom data, that can be used to access medical advice, or that can be used to assist with a diagnosis, must be developed as a ‘medical device’ ^16^. Might there be a requirement to think of software as an instrument that meets formal standards in other research settings ^17^? Would this make results more reliable, or would it stiﬂe creativity? Can we expect complete ‘precision’? If not, should there be ‘guards’ or ‘contracts’ to detail this? 

Software is not an oracle. There needs to be an improved understanding of which parts of a software tool can be treated as a black box and taken on faith, and which cannot. Without this understanding, software may be used in ways it is not designed for and so give spurious results. Software can be ﬂexible, and because of this, be used in domains for which it was not originally intended, and may not be appropriate; in this case, it should be validated within the new domain before any results are published.

**How does the research environment affect the translationprocess?** There is a perception that academic researchers are under pressure to publish at all costs, diminishing the attention paid to good software engineering practices, which are perceived as slowing down the research and publication process. Valuing software as a deliverable in its own right was viewed as an important part of improving its quality and availability. Citing software (via, e.g., the Journal of Open Source Software ^18^ or by more direct citations to the software ^19^) is another part of this process. Considering software explicitly as an output of research, and systematically assessing the impact of research software ^20, 21^, remains relatively unusual, and there is still work to be done in understanding how to achieve this. 

There was a view that funding bodies should be involved in discussions regarding theory-software translation. Many of the costs of software development, maintenance, and evolution are hidden; they need to be articulated, and be part of an open, ongoing conversation. The cost of developing software is often underestimated by principal investigators and funding bodies. A lot of time is spent porting software to new hardware, but it is difficult to obtain funding for this, with a negative impact on the quality of the software as a result.

**What is the best way to embed software engineering skills in science?** Often the people writing scientific code are graduate students or researchers who do not have a background in software engineering ^22, 23^. Data Carpentry/Software Carpentry was viewed as a good start, but not sufficient. Instilling the necessity of thinking about theory-software translation in graduate students right from the start would help to avoid the need to continually fix poorly-written and poorly-designed code. While this lack of training is a specific problem, software development training is a general challenge, because academic supervisors do not necessarily see the value of it, or even know about it themselves. Awareness that training exists, and a belief in the necessity of undertaking the training, is critical. There is potential for technical training to be conceptualised as a hierarchy, covering: the issues of theory-software translation at an abstract level; the principles of translating between theory and software at a process level; and in-depth expertise in the implementation of theory-software translation at the developer level (with possible specialization). 

Training in communication was also seen as essential, and should go both ways: all members of a research team need to be proficient in cross-disciplinary communication. Being able to communicate scientific requirements to software developers is essential. Being a careful and skeptical user of simulation outputs is also essential, and this requires an understanding of the workings and limitations of the software method, such that the outputs are viewed through the appropriate lens.

## Discussion

The material gathered from the workshop revealed a number of areas where theory-software translation research could significantly advance both knowledge and scientific practice. 

Supporting evidence for the identified themes comes from the Code/Theory workshop organised by authors CJ and RH in the UK, which examined the challenges faced by practising research software engineers and data scientists ^3^. The primary themes that emerged at the UK event related to designing software, sustaining software, and communication issues between domain scientists and software engineers, all of which map to our themes of research software *design*, *infrastructure*, and *culture*. The UK workshop focused on identifying practical solutions to these issues, with suggestions including improving communication, tools and training, and raising the profile of software in research, such that people understood its complexity and intellectual contribution. This last issue was also identified as a priority in a survey of Software Sustainability Institute Fellows, who comprise people who have been recognised for their contribution to the research software engineering community ^24^. 

Another activity that has provided insight into this area is the United States Research Software Sustainability Institute (URSSI) conceptualization project, funded by the US National Science Foundation. As part of this effort, the researchers conducted a survey of research software developers and users ^25^. Based on the 1,194 responses, they made some observations relevant to the results we report here. First, relating to our *Design* and *Culture* themes, the majority of respondents said they had not received software development training. Furthermore, only half of the respondents indicated sufficient training was available and only 25% said there was sufficient time for training. Second, regarding *Culture*, the vast majority of respondents indicated that the level of funding for research software was “insufficient and creates barriers to their work”. A wide-scale survey providing further strong evidence for the utility of theory-software translation research explicitly examined problems within research software engineering, providing a taxonomy of ‘pains’ covering technical, scientific and social issues ^26^. This work highlights that many common software engineering challenges, such as requirements gathering, communication difficulties and debugging, are particularly challenging— and often qualitatively different—within science, due to the nature of the research process, and the environment in which the work is conducted. 

Theory-software translation research has the potential to contribute to the evidence base for research software infrastructure strategy and practice at a local (individual/group), institutional (organization), national, and international level. It could also identify cross-cutting challenges for computational research, and advance the techniques we use to perform such research. We anticipate that a better understanding of the theory-software translation process will lead to more robust and accurate research software. 

The results of our analysis demonstrate that research software is not merely used to perform a task, but to understand a problem and advance knowledge. While current software engineering research outputs and methods are relevant to addressing these challenges, theory-software translation research would involve tackling new problems that are rooted within the scientific domain. We summarise key, emergent areas for research as follows:

- The translation process moves from theory to algorithm to software (and vice-versa). Information is lost in moving from one domain to another, as the way in which ideas are represented changes. Can we quantify or explain this loss/difference, and articulate the trade-offs resulting from translation?
- How does incorporating theory in software (e.g., a simulation) differ from standard requirements engineering? The development of software and theory happen together. While requirements changes are generally constrained for typical software, theory can change much more dramatically, resulting in not just an addition, but a fundamental divergence from what the software was initially designed to do.
- How do we understand the results of a simulation, and translate this back to the underlying theory? How should real world data be used in the verification process?
- How do we go from viable theory to validated, verified code in a time-efficient way? Can theory be expressed as a set of tests?
- There is a distinction between theory, model, numerics, and code, and there are difficulties mapping between them. There can be errors in any part of the mapping process that may affect the resulting science. How can we detect and handle these errors?
- Is a true separation of concerns (theory, and its implementation in software) possible? What are the implications for how we write scientific software?
- Should theory be recoverable from software (where software includes documentation)? Can theory today be represented solely in papers, or is it really also in the code? How can we help people to read and understand it?
- The increase in model complexity and sophistication of questions that models are designed to answer makes translating them into software increasingly difficult. How do we determine the appropriate level of complexity? Is it possible to optimise for complexity reduction, as well as performance?
- How can we define functional reproducibility? Long-term reproducibility is likely to remain out of reach due to software collapse ^13^. Can we create ways of representing theory that will persist and remain usable, so as to increase software sustainability and prolong the period of reproducibility? Is there a way of providing a reference implementation that can be used as a blueprint for the theory?
- Can we identify the range of practices currently used, and gather empirical data concerning their efficacy, to result in evidence-based best practice?
- What are the benefits and implications of the increasing use of automation in research software engineering?
- Software is unlikely to ever be 100% ‘correct’. Can we measure how well software represents theory, and estimate the error that might have been introduced during the translation process? Can we develop ways to quantify uncertainty for software functioning such that appropriate probabilities can be applied to results?

## Conclusions

The discussion sessions at the theory-software translation workshop identified a series of challenges in building and using scientific software. Related research questions map to the areas of research software *design*, *infrastructure*, and *culture*. Whilst the distinctions between these categories are not absolute, they provide a means of structuring our understanding of the theory-software translation process, and advocating for its improvement via research.

## Data availability

The workshop documents have all been retained in their original form, but they are highly identifying (including the names of all individuals who contributed to and commented on them, as well as personal views and experiences). Formal IRB approval was not sought for the study, as we instead used the collective ownership model described in the Methods section. We do not have explicit permission from workshop participants to share the original documents. 

Emails between the authors, and iterative refinement of the manuscript draft on the Overleaf platform also formed part of the analysis process, and could be viewed as contributing additional data/metadata. We would be happy to answer questions on the data and analysis process. All queries should be directed to the corresponding author, CJ. The validity of the results is guaranteed in that they are co-produced and represent the cumulative, collective reﬂections of the authors, as they see them, based on the workshop discussions. The workshop report ^4^ can be considered as a published, interim stage in the analysis process of moving from the discussion documents to this final output.
