# Supplementary material for: The challenges of theory-software translation
Source: F1000Res. 2020 Oct 2;9:1192. [Version 1] doi: 10.12688/f1000research.25561.1 (PMC7656273; doi:10.12688/f1000research.25561.1)
Supplement: Peer review history

# Peer review: Reviewer response for version 1

**DOI:** 10.5256/f1000research.28209.r72412

**Published:** 2020-11-10

**Authors:** Mozhgan Kabiri Chimeh

**Affiliations:** 1 NVIDIA, UK, Sheffield, UK

The abstract and title reflect the content of the article. The motivation and contribution of the paper are clear. It’s well written. A good range of papers were included in the related work paper.

 Very good, comprehensive workshop report that can be used as a good base for future similar events for further discussion.

 My only negative feedback is for the workshop itself. Looking at the speakers' list, I can hardly see a diverse and inclusive list! That is something that is missing really, and I hope in future events and workshops as such could be kept as diverse and inclusive as possible to give stronger meaning to the results and conclusions.

Is the work clearly and accurately presented and does it cite the current literature?

Yes

If applicable, is the statistical analysis and its interpretation appropriate?

Not applicable

Are all the source data underlying the results available to ensure full reproducibility?

Yes

Is the study design appropriate and is the work technically sound?

Yes

Are the conclusions drawn adequately supported by the results?

Yes

Are sufficient details of methods and analysis provided to allow replication by others?

Yes

Reviewer Expertise:

High-performance computing. Simulation acceleration. Optimization and tuning of scientific applications.

I confirm that I have read this submission and believe that I have an appropriate level of expertise to confirm that it is of an acceptable scientific standard.

# Peer review: Reviewer response for version 1

**DOI:** 10.5256/f1000research.28209.r72420

**Published:** 2020-10-26

**Authors:** Mike Heroux

**Affiliations:** 1 Sandia National Laboratories (SNL), Albuquerque, NM, USA; 2 St. John's University, Collegeville, MN, USA

This article represents a summary and analysis of the output from the 2019 Theory-Software Translation workshop held in New Orleans, LA, USA in February 2019. 

 The authors summarize the purpose and format of the workshop and then present an analysis of the workshop breakout session content, folding the content into a holistic discussion that characterizes the challenges of theory-software translation, key research questions, and some discussion of potential solution strategies.

 Overall, the article is very well written. The authors are some of the leaders in this field, which is reflected in the scope of discussion. I have just a few comments and suggestions:

- While this article is about theory-software translation, did the topic of model-free approaches come up during the workshop or subsequent discussions? For example, some scientific teams are successfully using machine learning (ML) approaches, where prediction and insight may come from an inference engine constructed from raw data with no explicit mathematical model. These approaches are increasingly integrated into more established theoretical frameworks, with promising impact. If there were no comments in the workshop, then omitting the topic from this article is appropriate.
- In the introduction, you state that “Validation of the artefact—being sure it is a true representation of the theory—is even more challenging.” Is this a sufficiently expansive definition of validation? The terse definition of validation as “Doing things right.” is often interpreted as meaning that the computational results are consistent with physical experiments, not just the theory that the software encodes.
- You state that the workshop discussion notes were coded. To a software person who is not familiar with text coding, this term may be unfamiliar, and may indeed be confused by their familiarity with software coding. A brief footnote, parenthetic explanation, or similar explanation could improve the accessibility of the paper, since the coding activity was important for producing the article content.
- At the end of page 4, you state, “Ince et al. argue for the necessity of source code provision along with papers, citing research showing poor effectiveness of specifications in producing equivalency across implementations.” I think this sentence could be expanded and better explained for the reader. In particular, what are the definitions of provision, specification, and equivalency? I think I understand the statement, but a simple example might help the reader.
- While I have no concerns about the validity of the content in this article, the unavailability of the raw workshop discussion notes and the informal nature of the coding process used on the combined discussion notes text, prohibits rigorous reproducibility of the results. The omission is clearly acknowledged and explained, so I do not view it as a critical problem. Perhaps future approaches could be designed to permit availability of raw content by anonymizing attribution or getting ARB approval. Automated, or carefully documented coding would be useful too.

Is the work clearly and accurately presented and does it cite the current literature?

Yes

If applicable, is the statistical analysis and its interpretation appropriate?

Not applicable

Are all the source data underlying the results available to ensure full reproducibility?

No

Is the study design appropriate and is the work technically sound?

Yes

Are the conclusions drawn adequately supported by the results?

Yes

Are sufficient details of methods and analysis provided to allow replication by others?

Yes

Reviewer Expertise:

Scientific software, high-performance computing, mathematical modeling and simulation

I confirm that I have read this submission and believe that I have an appropriate level of expertise to confirm that it is of an acceptable scientific standard.

# Peer review: Reviewer response for version 1

**DOI:** 10.5256/f1000research.28209.r72416

**Published:** 2020-10-14

**Authors:** Konrad Hinsen

**Affiliations:** 1 Center for Molecular Biophysics, CNRS, Orléans, France; 2 Synchrotron SOEIL, Saint Aubin, France

This article presents a structured summary of the discussion sessions of a recent workshop on theory-software translation, followed by carefully crafted arguments for establishing theory-software translation as a new field of research. It represents a significant effort at making progress on an important question, but I don't consider it a "research article" for the reasons outlined below, and therefore I can approve it only with reservations. My recommendation to the authors is to relabel their work as an opinion article.

 A research article should start with a research question, and my first task as a reviewer would be to judge if the method design is appropriate to answer this question. Indeed, a question in the review form is "Is the study design appropriate and is the work technically sound?" In the absence of a research question, I cannot answer it meaningfully. One can imagine an underlying research question such as "Which issues do practicing scientists and engineers see with the current state of theory-software translation?", and then the method design could be evaluated critically (for example, I would criticize the overrepresentation of HPC practitioners, and the complete absence of theoretical scientists not involved with software development). But then, the call for action in the final sections would be inappropriate and would have to be replaced by something like "We find that practitioners believe that...". Put differently, you can study science policy or shape science policy, but you cannot do both at the same time. My remaining comments on this article assume that its main intention is to shape science policy.

 The authors state the goal of the proposed new research area of theory-software translation as "investigating how software is constructed and its outputs used within science". This indicates a strong focus on the software end of the bridge being envisaged. In my opinion, it is also necessary to investigate how theoretical work is being performed in science, how computers have impacted the practices of theoreticians, and how their use of computers could be improved in the future. This would lead to a stronger focus on non-numerical computational tools, for example, computer algebra systems, which I expect to be important in improving theory-software translation. It would also help with answering some of the questions raised in the workshop. The answer to "Should theory be readable from software?" is clearly "yes" for computer algebra applications. And that means that if we succeed in creating formally verifiable correspondences between computer algebra and high-performance simulation software, the readability of the latter is no longer an issue.

 It would also be useful to separate the challenges and goals of theory-software translation research into short-term and long-term. Short-term work, which is what the article seems to concentrate on, must take into account the constraints of existing technology that cannot be overcome quickly. Long-term work can be more ambitious and envisage completely new software and tool stacks designed specifically for supporting scientific research. Importantly, long-term research is necessary to detect if short-term efforts are likely to create additional technical debt that then becomes an obstacle to more substantial progress.

 There is one sentence in the article that I strongly disagree with: "Whilst theory is often exact, code has tolerances and approximations." (page 6). Approximations are an important aspect of all theoretical work in the natural sciences. Exact theory can only exist in what Herbert Simon called the "sciences of the articifial". In particular, the approximations made in simulation and data analysis, for example, numerical approximations, clearly belong to the domain of theory, not software. The best evidence is that such questions are discussed in research articles on numerical algorithms, independently of any concrete implementation. In the final list of research questions for the new field, the one starting with "There is a distinction between theory, model, numerics, and code" is related to this point and suggests that the authors are mainly considering the scientific disciplines represented in HPC applications. There are disciplines that do not have theories as frameworks for their models or use models that are not numerical.

 The section on "Measuring uncertainty in theory-software translation" (page 6) overlooks the fundamental lack of robustness in software. Unlike in many other domains of engineering, there is no way to ensure that small mistakes have small consequences (see https://dx.doi.org/10.1109/MCSE.2016.67 for a detailed discussion) ^1^. As a consequence, the "x% chance some error has been introduced along the way" is of little practical relevance because it permits no conclusion about the possible magnitude of the resulting error in the output produced by the software. Long-term work on theory-software translation should therefore investigate paths to higher robustness. For example, do we really need Turing-complete languages to implement scientific models? Maybe a less powerful medium could help reduce the impact of errors.

 Finally, a minor technical point: "such as the representation of real numbers" (page 6) suggests that real numbers can be represented in a computer, whereas the root issue with precision-related problems is that a finite-size representation for arbitrary real numbers doesn't exist. The largest subset of the real numbers that are representable in a computer is the set of computable numbers, which were first studied by Alan Turing in his seminal paper introducing the Turing machine.

Is the work clearly and accurately presented and does it cite the current literature?

Yes

If applicable, is the statistical analysis and its interpretation appropriate?

Not applicable

Are all the source data underlying the results available to ensure full reproducibility?

Yes

Is the study design appropriate and is the work technically sound?

No

Are the conclusions drawn adequately supported by the results?

Partly

Are sufficient details of methods and analysis provided to allow replication by others?

Partly

Reviewer Expertise:

scientific computing, statistical physics, biomolecular simulation, protein dynamics

I confirm that I have read this submission and believe that I have an appropriate level of expertise to confirm that it is of an acceptable scientific standard, however I have significant reservations, as outlined above.

## References

[1] : The Power to Create Chaos. *Computing in Science & Engineering*.2016;18(4) : 10.1109/MCSE.2016.67 75-79 10.1109/MCSE.2016.67 29033693
